# A case of pulmonary visceral subpleural hematoma treated by hematoma evacuation during care of post-cardiopulmonary resuscitation

**DOI:** 10.1186/s13019-024-02769-w

**Published:** 2024-04-20

**Authors:** Yutaka Funaki, Kyoji Hirai

**Affiliations:** 1 Shock and Trauma Center, Nippon Medical School, Chiba Hokusoh Hospital, 1715 Kamagari, Inzai, Chiba, 270-1694 Japan; 2 Department of Thoracic Surgery, Nippon Medical School, Chiba Hokusoh Hospital, 1715 Kamagari, Inzai, Chiba, 270-1694 Japan

**Keywords:** Visceral subpleural hematoma, CPR, Chest compression, Anticoagulant, Antiplatelet

## Abstract

**Background:**

The occurrence of pulmonary visceral subpleural hematoma during care of post-cardiopulmonary resuscitation including chest compressions and anticoagulant and antiplatelet therapies is extremely rare. Also, there are few reports of treatment of visceral subpleural hematoma, most of which are treated by lung resection. Here we describe a rare case that pulmonary visceral subpleural hematoma arose during post-cardiopulmonary resuscitation care and was treated by hematoma evacuation.

**Case presentation:**

A 58-year-old male with no smoking history and, past medical histories of rheumatoid arthritis, chronic atrial fibrillation, hypertension, diabetes, and dyslipidemia developed ventricular fibrillation due to myocardial infarction and fainted. He received bystander cardiopulmonary resuscitation and defibrillation by the ambulance crew and had return of spontaneous circulation. After transfer to our hospital, the patient underwent percutaneous catheter intervention and stenting with a diagnosis of myocardial infarction, followed by anticoagulant and antiplatelet therapies. On the 8th hospital day, chest radiography suggested right lower lobe pneumonia, and subsequent chest computed tomography revealed pulmonary hematoma in the visceral subpleural area from S6 to S10. Since no improvement was observed in hypoxemia, treatment was considered necessary. First, an attempt at computed tomography-guided drainage of hematoma was made, but insertion of the Pig-tail catheter was difficult due to hardness of the hematoma. Next, evacuation of hematoma was performed on the 13th hospital day. The hematoma was located in the visceral subpleural area and was removed by incising the pleura. TachoSil Tissue Sealing sheet and Polyglycoal acid sheet were applied to the sites of air leakage and oozing after hematoma evacuation. No re-bleeding or air leakage was observed after the treatment, and the patient was discharged on the 26th hospital day after an uneventful course.

**Conclusions:**

Pulmonary visceral subpleural hematoma may occur during post-cardiopulmonary resuscitation care, including chest compressions and anticoagulant and antiplatelet therapies. In our case, CT-guided puncture and drainage was difficult and surgical treatment by incision of the visceral pleura and hematoma evacuation alone was done successfully.

## Background

Cardiopulmonary resuscitation (CPR) including chest compression is an essential procedure for cardiopulmonary arrest, but it may cause chest trauma, such as rib fracture, sternal fracture, pulmonary contusion, and hemothorax, and injuries of the abdominal organs such as the liver and spleen [1]. After return of spontaneous circulation, the primary disease must be treated promptly. In the case of acute myocardial infarction, immediate revascularization and subsequent antiplatelet therapy is required.

Intrapulmonary hematoma is caused primarily by chest trauma, but reports of visceral subpleural hematoma are small in number [2]. And there is as of yet no report of visceral subpleural hematoma during post-CPR care. Also, while intrapulmonary hematoma usually disappears without treatment, drainage may be necessary if the hematoma enlarges or there are signs of infection. We report a case that showed enlargement of visceral subpleural hematoma during care of post-cardiopulmonary resuscitation including chest compressions and anticoagulant and antiplatelet therapies underwent evacuation of it. Reports of treatment of pulmonary visceral subpleural hematoma are still small in number, and there is room for discussion.

## Case presentation

The patient was a 58-year-old male with no smoking history and histories of rheumatoid arthritis, chronic atrial fibrillation, hypertension, diabetes, dyslipidemia. He had been prescribed including anticoagulants, rivaroxaban. He suddenly fainted in the presence of colleagues and promptly received bystander CPR by colleagues. The ambulance crew arrived after 10 min and performed defibrillation once after checking the VF waveform. Return of spontaneous circulation (ROSC) was achieved soon, and the patient was transported to the hospital that first treated him. At the time of arrival, ECG showed atrial fibrillation, ST elevation in aV_R_, and diffuse ST depression in V1-6, suggesting cardiopulmonary arrest due to ST-elevation myocardial infarction (STEMI), and the patient was transferred to our hospital.

At the time of the arrival at our hospital, the patient had tracheal intubation and showed a heart rate of 124 bpm, blood pressure of 148/79 mmHg, 100% arterial blood oxygen saturation by 10 L/min oxygen administration, body temperature of 36.5 °C, and a state of consciousness of GCS E1V1M4 under midazolam sedation. Aspirin and prasugrel were administered, and coronary angiography (CAG) was performed. The results showed that 99% of stenoses were observed in #6–7 and #9, and, with a diagnosis of STEMI, percutaneous catheter intervention (PCI) and stenting were performed. Intravenous administration of heparin was initiated during PCI. Also, while computed tomography (CT) performed at the time of arrival showed bilateral pleural effusion and pneumonia with atelectasis on the dorsal side, no rib fracture and no clearly intrapleural hematoma formation was noted.

The patient was thereafter managed in the intensive care unit(ICU). Induced hypothermia with a target temperature of 34 °C was performed for 24 h, followed by rewarming over 24 h. As for anticoagulant therapy instead of revaroxaban, activated clotting time was controlled at ≥ 250 s by intravenous administration of heparin Na, and the administration of aspirin and prasugrel was continued. For pneumonia, ABPC/SBT was administered for 3 days, followed by PIPC/TAZ. The patient was extubated on the 7th hospital day as the extubation criteria were met. No bleeding into the airway was noted during the course.

**Fig. 1:**
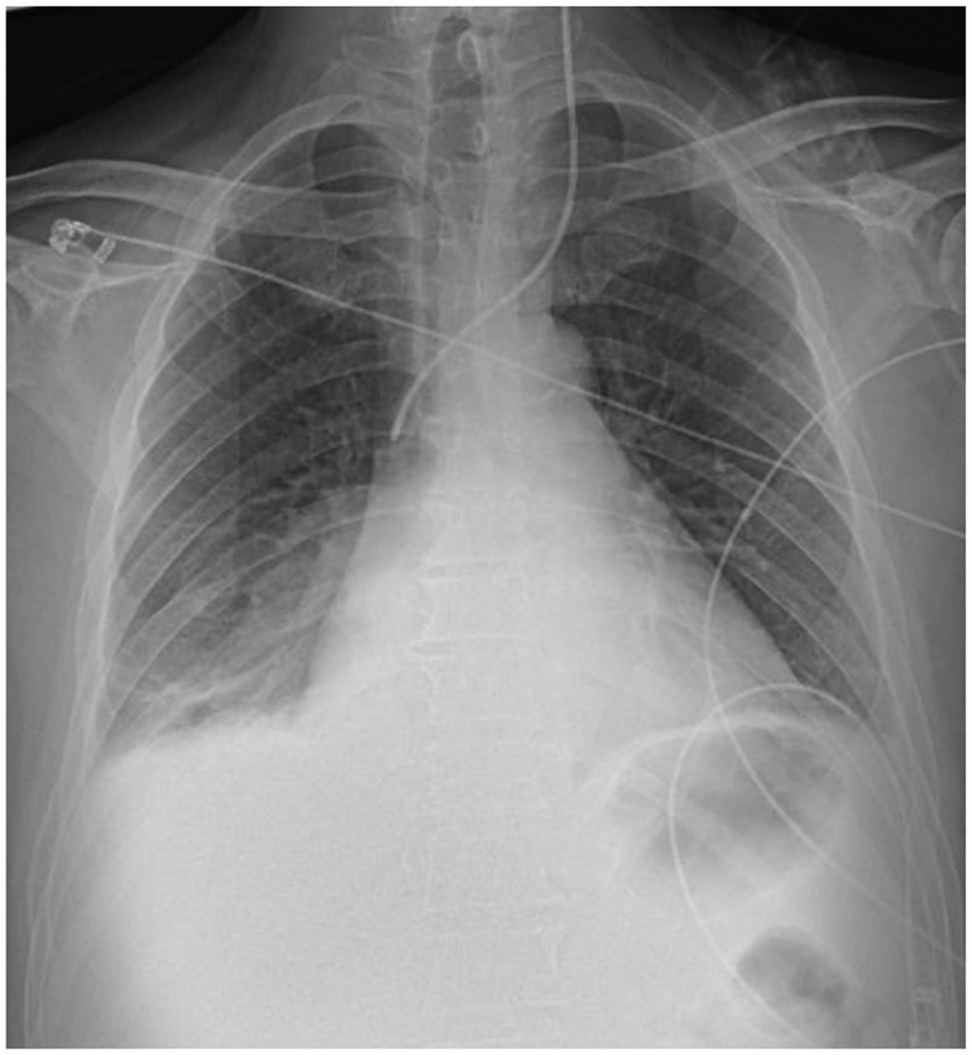
Plain chest X-ray findings: Hypolucency is noted in the right lower lung field

**Fig. 2:**
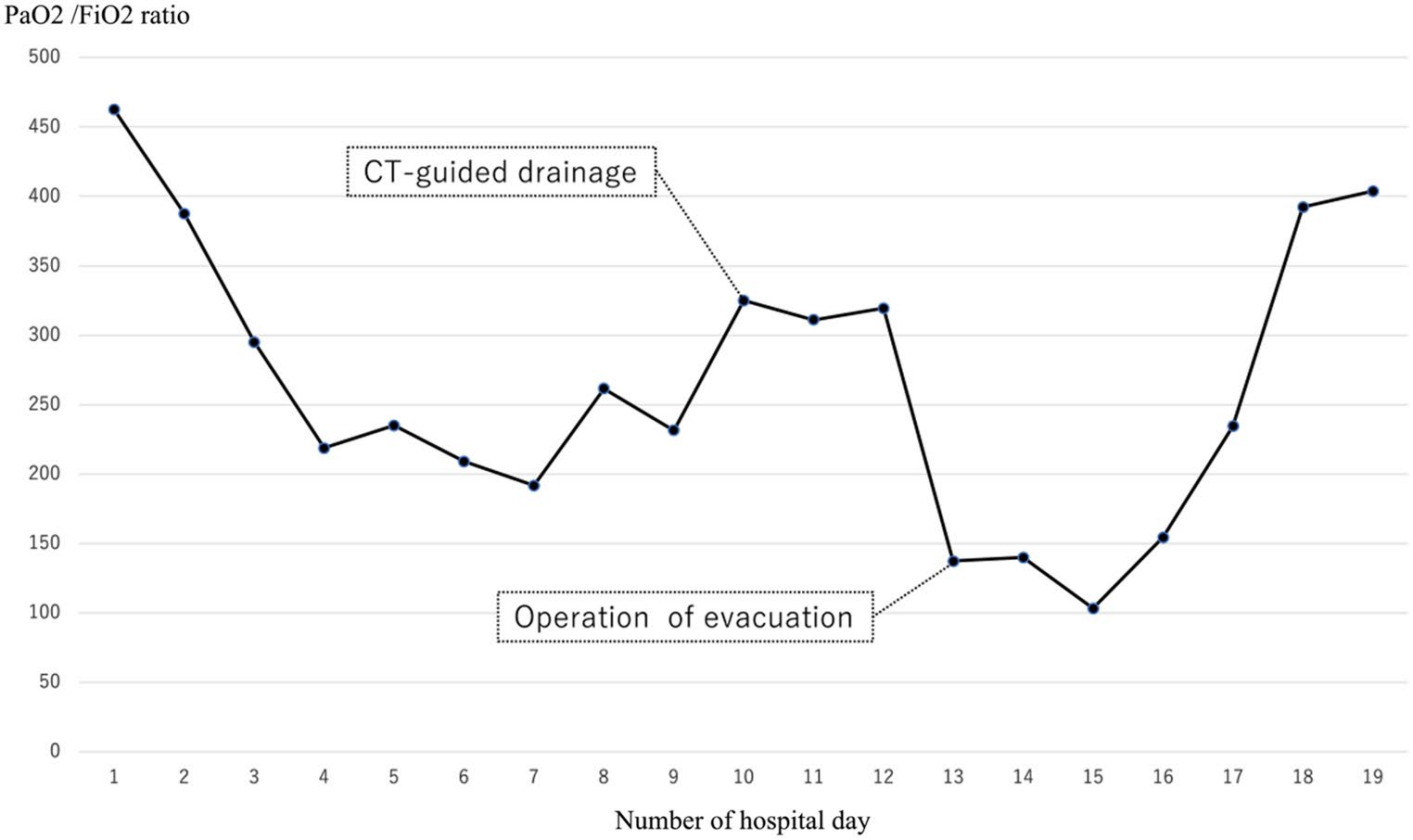
The graph of PaO2 /FiO2 ratio and oxygen intervention from admission to ICU discharge

**Fig. 3:**
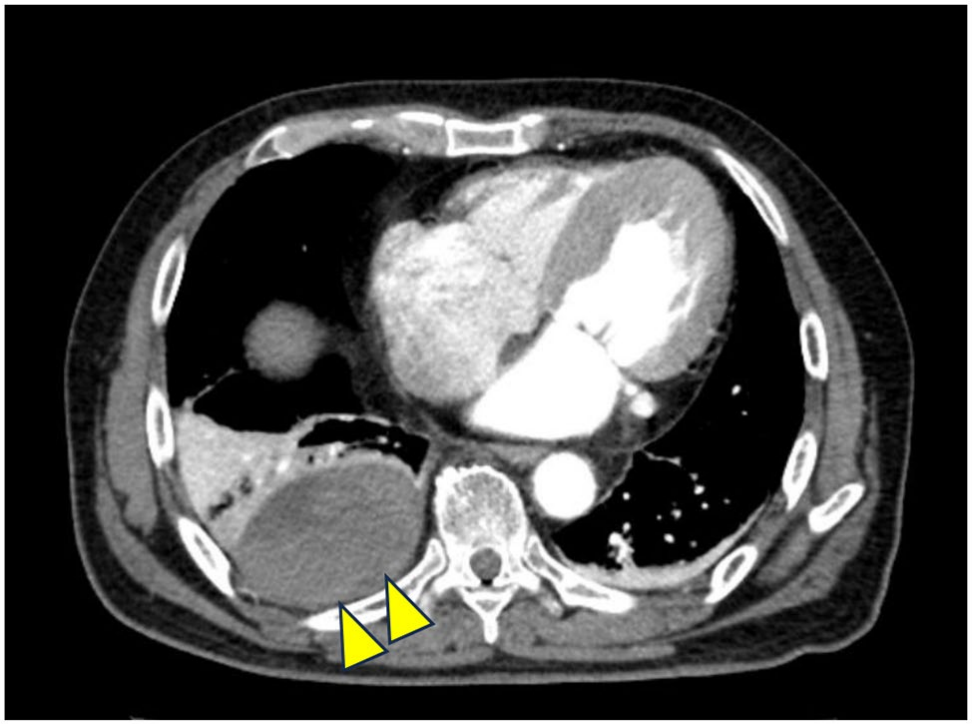
Chest CT findings: Hematoma 72 mm×52 mm×103 mm was observed immediately below the visceral pleura on the dorsal side of the right lower lobe. (yellow arrows)

Heparin administration was ended on the 8th hospital day and was switched to rivaroxaban. On the same day, radiolucency was found to be reduced in the right middle and lower lung fields on chest radiography, and pneumonia and pleural effusion were suspected (Fig. 1). Although antibiotics and diuretics were used for the treatment, the response of the respiratory condition was poor with PaO2 /FiO2 ratio = 231 (Fig. 2). Therefore, to examine the status of pneumonia and pleural effusion, CT was performed on the 9th hospital day, disclosing the presence of intrapulmonary hematoma (size:72 mm×52 mm×103 mm) on the dorsal side of the right lower lobe (Fig. 3).

Since little improvement of respiratory state was observed also on the 10th hospital day, we decided to remove this hematoma. CT-guided puncture and drainage of the hematoma in the right lower lobe were attempted, Because echography showed uncertain boundaries between hematoma and lung parenchyma, Although the guide wire could be inserted, insertion of the pig-tail catheter was difficult, and drainage could not be achieved. This procedure induced pneumothorax, and the fluid discharged into the thoracic cavity was yellowish and serous, indicating that the lesion was intrapulmonary hematoma. Since CT-guided drainage was difficult, surgical treatment was selected, and surgery was scheduled on the 13th hospital day.

**Fig. 4:**
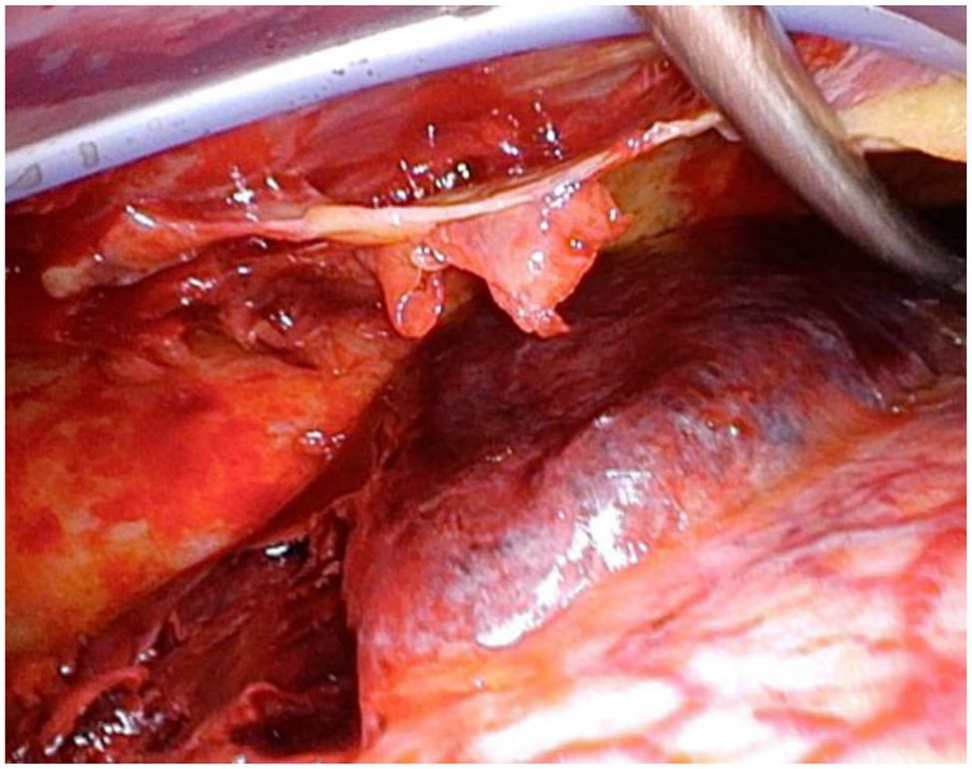
Intraoperative findings: Hematoma was present on the dorsal side of the lower lobe of the right lung. It was removed by pleural incision

**Fig. 5:**
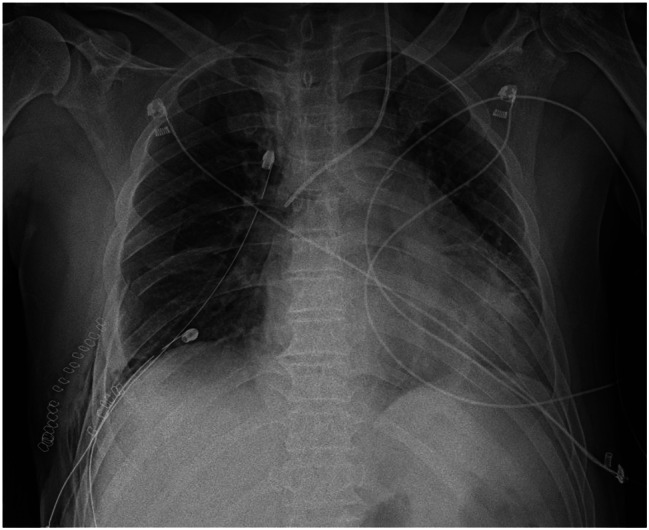
Plain chest X-ray findings: Radiolucency of the right lower lobe was markedly improved

Thoracoscopically assisted evacuation of hematoma in the lower lobe of the right lung was performed. Thoracotomy was made at the right 5th intercostal space. Thoracoscopy showed visceral subpleural hematoma on the dorsal side of S6-10 of the right lung. The visceral pleural was incised, and 50 g of hematoma was resected (Fig. 4). To the area where slight air leakage and oozing were observed, TachoSil Tissue Sealing sheet was applied, and Polyglycoal acid (PGA) sheet (5 × 10 cm) was attached for additional strength. Surgery was ended by placing 24Fr double-lumen thoracic drains on the diaphragm and dorsal aspect of the lung. The operative time was 125 min, and the volume of hemorrhage was 200 mL. On chest radiography taken just after operation, radiolucency of the right lower lung field was markedly improved compared with the preoperative images (Fig. 5).

**Fig. 6:**
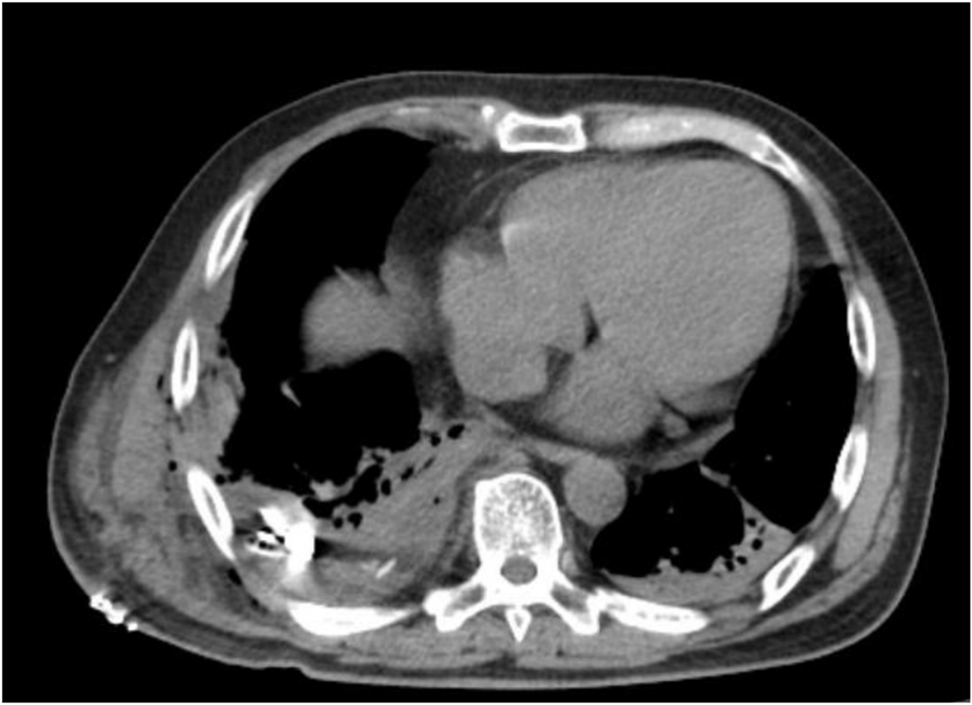
Chest CT findings: Most of the hematoma has been removed. Artifacts from TachoSil Tissue Sealing sheet (CSL Behring) and Polyglycolic acid sheet were visible

Although the respiratory condition temporarily worsened after surgery, CT showed hematoma almost removed and no cause for exacerbation (Fig. 6). It gradually improved from 3 days after operation. The first drain on the dorsal side was removed on the 16th hospital day. PIPC/TAZ had been completed on the 17th hospital day. The respiratory condition was improved to PaO2 /FiO2 ratio = 403 on the 19th hospital day (Fig. 2), and the patient was discharged from the ICU. The another drain was removed on the 20th hospital day. Thereafter, cardiac rehabilitation was implemented, and the patient was discharged to home with no disabilities on the 26th hospital day. He has returned to society without any particular complications during the two-year period.

## Discussion

The case presented here is pulmonary visceral subpleural hematoma, in a patient who exposed to chest compressions during CRP interventions, was detected during anticoagulant and antiplatelet therapy. Pulmonary visceral subpleural hematoma is an intrapulmonary hematoma located immediately below the visceral pleura. Intrapulmonary hematoma are reported to be caused mostly by trauma, but there are few reports about pulmonary visceral subpleural hematoma, with causes varying from pressure changes due to movements of the diaphragm such as cough, anticoagulant therapy [2] and bleeding into bullae [3]. Chest compressions during CPR, by its own energy can cause complications, including chest trauma [1]. This patient was underwent CPR for more than 10 min, which would have taken enough for a visceral subpleural hematoma to form. Therefore, we suspected that chest compressions during CPR is one of the causes of the visceral subpleural hematoma. Actually, the energy from chest compressions must be added from the front, but it is doubtful that visceral subpleural hematoma was formed dorsally. However, similar to brain injury, lung injury has coup and contre-coup lesions. Dorsal hematomas can be explained by contre-coup lesions. If anterior energy from sternal compression compresses the lung, causing it to collide with the dorsal thorax, injury occurs to the dorsal lung as contre-coup lesions. Fortunately, there were no rib fracture or bleeding into the airway during artificial respiratory management.

Antiplatelet and anticoagulant therapies were indispensable in this case, because prompt stenting was necessary for the management of myocardial infarction. There are reports that anticoagulant therapy is a risk factor of hematoma development [2, 3]. Antiplatelet and anticoagulant therapies are also considered to have been involved in hematoma development. We assume that the small visceral subpleural hematoma, which was unnoticed at the time of arrival, was enlarged by antiplatelet and anticoagulant therapies and finally became apparent on the 9th hospital day. In addition, an involvement of vascular vulnerability due to underlying diseases such as chronic rheumatoid arthritis and diabetes cannot be excluded.

Intrapulmonary hematoma is reported to usually remain asymptomatic and, without an increase in bleeding or infection, disappear in several to 6 months [4]. If it is expected to be complicated by bleeding or infection, surgery is necessary [2, 3], and there have also been undiagnosed cases for which diagnostic treatments were selected [5]. In the present case, intervention by procedures such as drainage was considered necessary from enlargement of hematoma during antiplatelet and anticoagulant therapies in a relatively short period after the responsible trauma and persistence of hypoxemia.

For the surgical treatment of pulmonary visceral subpleural hematoma, procedures such as CT-guided drainage and surgical evacuation of hematoma are selected [2, 3, 5]. In the present case, less invasive CT-guided drainage of hematoma was attempted first. Echo-guided drainage was considered, but could not be performed because the boundary between the hematoma and the lung was unclear. However, while the guide wire could be inserted, the catheter was difficult to insert probably because of the hardness of the hematoma contents. Indeed, one cases in which drainage by CT-guided pleural puncture was difficult have been reported [2].

Procedures including wedge resection, segmentectomy, evacuation of hematoma by pleural incision, and running suture of the defect after evacuation of hematoma have been reported as surgical treatments for visceral subpleural hematoma [2, 3, 5]. Since the hematoma in our case extended over a wide area in the right lower lobe, we treated it by open chest evacuation of hematoma and reinforcement with TachoSil Tissue Sealing sheet (CSL Behring) and Polyglycolic acid sheet alone in expectation of recovery of pulmonary function after evacuation of hematoma. Plication of the lung by running suture was considered to close the space after evacuation of hematoma and incised visceral pleura. But suturing of this site was avoided in consideration of the absence of signs of infection, vulnerability of lung tissue due to inflammation, necessity of postoperative anticoagulant and antiplatelet therapies and the consequent likelihood of the development of similar hematomas under the visceral pleura in the future, and the possibility of insufficient lung inflation after recovery of pulmonary function. The postoperative course was uneventful without air leakage or bleeding. It was suggested that evacuation of hematoma by pleural incision and application of reinforcement materials to the defect suffice as treatment for hematomas immediately below the visceral pleural on condition that there are no signs of infection.

In this case presentation, respiratory condition did not improve promptly after surgery. The worsening of PaO2 /FiO2 ratio over 3days after operation was thought to be due to surgical invasion and anesthesia with single lung ventilation. Therefore, it is unclear whether the surgery contributed to a rapid improvement in respiratory condition, a shorter ICU stay, or prevention further hematoma development. However, with careful consideration of residual lung findings after hematoma removal, this procedure without performing lung resection is an option of minimally invasive surgery for patients in such condition.

## Data Availability

All datasets generated or analyzed during this study are included in this published article.

## Abbreviations

CPR: cardiopulmonary resuscitation
ROSC: Return of spontaneous circulation
STEMI: ST-elevation myocardial infarction
CAG: coronary angiography
PCI: percutaneous catheter intervention
CT: computed tomography
ICU: intensive care unit
